# Evaluation of a mobile mammography unit: concepts and randomized cluster trial protocol of a population health intervention research to reduce breast cancer screening inequalities

**DOI:** 10.1186/s13063-022-06480-w

**Published:** 2022-07-08

**Authors:** Elodie Guillaume, Quentin Rollet, Ludivine Launay, Séverine Beuriot, Olivier Dejardin, Annick Notari, Elodie Crevel, Ahmed Benhammouda, Laurent Verzaux, Marie-Christine Quertier, Guy Launoy

**Affiliations:** 1 U1086 INSERM “ANTICIPE” Caen Normandy University - Equipe Labellisée Ligue, Contre le Cancer, Caen, France; 2 Centre de lutte contre le cancer François Baclesse, Caen, France; 3 CHU CAEN, Caen, France; 4 DIS ORNE, Alençon, France; 5 Centre Régional de Coordination des Dépistages des Cancers Normandie, Caen, France

**Keywords:** Breast cancer screening, Mobile mammography, Social and territorial inequalities, Randomized cluster trial, Population health intervention research

## Abstract

**Background:**

Breast cancer is the leading cancer in women in France both in incidence and mortality. Organized breast cancer screening (OBCS) has been implemented nationwide since 2004, but the participation rate remains low (48%) and inequalities in participation have been reported. Facilities such as mobile mammography units could be effective to increase participation in OBCS and reduce inequalities, especially areas underserved in screening. Our main objective is to evaluate the impact of a mobile unit and to establish how it could be used to tackle territorial inequalities in OBCS participation.

**Methods:**

A collaborative project will be conducted as a randomized controlled cluster trial in 2022–2024 in remote areas of four French departments. Small geographic areas were constructed by clustering women eligible to OBCS, according to distance to the nearest radiology centre, until an expected sample of eligible women was attained, as determined by logistic and financial constraints. Intervention areas were then selected by randomization in parallel groups. The main intervention is to propose an appointment at the mobile unit in addition to current OBCS in these remote areas according to the principle of proportionate universalism. A few weeks before the intervention, OBCS will be promoted with a specific information campaign and corresponding tools, applying the principle of multilevel, intersectoral and community empowerment to tackle inequalities.

**Discussion:**

This randomized controlled trial will provide a high level of evidence in assessing the effects of mobile unit on participation and inequalities. Contextual factors impacting the intervention will be a key focus in this evaluation. Quantitative analyses will be complemented by qualitative analyses to investigate the causal mechanisms affecting the effectiveness of the intervention and to establish how the findings can be applied at national level.

**Trial registration:**

Registered on ClinicalTrials.gov, December 21, 2021: NCT05164874.

## Introduction

### Background

Cancer remains the leading cause of death and a pathology where health inequalities are particularly marked. These inequalities are evident at all stages of the medical history of the disease and are revealed with indicators such as incidence, survival and mortality. Regarding detectable cancers like breast cancer, screening is a key step in the construction of inequalities [1]. In most European Union member states, breast cancer screening is based on regular mammography screening with some differences in implementation [2]. In France, breast cancer is the leading cancer in women in incidence and mortality with 58,968 new cases estimated for 2017 and 11,883 deaths in the same year [3]. Mass screening has been organized nationwide since 2004 according to European recommendations, with high quality assurance. A screening mammography is offered every two years to women aged 50 to 74 with an average risk. Women are invited by management structures in charge of this screening (SMS) to visit an accredited radiologist’s office where radiological imaging of the breast (two views) and a clinical breast examination are performed. The radiologist interprets the images. If the images are normal, a second radiologist reviews them. In the last three years, the national participation rate in organized breast cancer screening (OBCS) has remained stable around 48% [Santé Publique France], while the rate of opportunistic screening with attendance is estimated at 15%. However, the European Union recommendation is 70% [4].

Few literature reviews on OBCS have been conducted to identify common determinants of non-adhesion to it, yet the organization of OBCS, its screening modalities and targeted populations (low income, ethnic group, rural areas) differ between countries [5, 6]. Consensually established determinants are usually grouped in five categories: (a) socio-demographics characteristics such as age, marital status, low income; (b) environmental characteristics like living in rural areas [7]; (c) health system utilization: having a general practitioner; (d) health behaviour such as performing other types of screening, alcohol or tobacco consumption and (e) psychological factors such as beliefs and concerns. Most of these factors are socially determined, and social inequalities in participation in OBCS are frequently reported. In France, participation rates vary greatly, some departments having a participation rate close to the European benchmark and others having a very low rate (< 25%). By using ecological indexes of deprivation [8] and small geographical units, social and territorial inequalities have been highlighted with a low participation for women living in deprived areas or far from a radiologist’s office [9–12].

*The World Health Organization* (WHO) considers social determinants to be the main causes of inequalities in health. These are the circumstances in which people are born, grow up, live, work and age and the systems set up to deal with disease. These determinants are multiple, ranging from individual to global, and they interact in complex ways. Determinants of non-adhesion to OBCS can also be established by using social ecological models that incorporate all the social and ecological factors that affect breast screening participation [13]. In summary, demographic, socio-economic determinants and health behaviours concern individuals, social support and social networks have an interpersonal dimension, and cultural norms and community organization involve issues pertaining to the community. Another dimension is the health care system, which involves factors such as health insurance, travelling distance to health facilities. Finally, there are structural factors relating to inequalities in wealth and place of residence. When modifiable, these determinants can be the target of interventions to increase OBCS participation and reduce inequalities in participation.

### Rationale

Various strategies can be implemented to increase participation in OBCS in deprived populations or in remote areas. Among these, mobile mammography units (MMUs) are currently operating in many areas in the world : USA [14], Brazil [15], and at least 7 countries in European Union [16]. Depending on the country, MMUs are included in the national program of organized cancer screening (Sweden [17], or in regional programs [10, 14, 18], in addition to the national screening program. MMUs are considered to increase access to OBCS for under-screened groups by increasing physical and economic access while reducing barriers for women like structural barriers and out-of-pocket costs. However, very few studies have provided robust evidence-based data through an RCT. Moreover, regardless of their target, most evaluations have not taken the existence of proven inequalities into account in their design and have not set out to reduce social and territorial inequalities in participation as an objective [19].

In France, a retrospective study conducted in 2019 in a rural area suggested that, when used in remote areas, MMUs can reduce social and territorial inequalities [10]. However, the potential value of MMUs depends on socio-demographic, geographical and medical characteristics. Therefore, there is very little evidence to help public health decision-makers in France to organize OBCS that aims to reduce health socio-territorial inequalities.

### Objectives and trial design

Only an experiment rigorously conducted and evaluated over a large territory would provide reliable information on the effectiveness of an MMU to increase participation and reduce social and territorial inequalities, with a view to a subsequent national rollout. We thus set up a prospective randomized controlled cluster trial in the general population with parallel group superiority trial and 1:1 allocation ratio for clusters to assess the gain in participation obtained with an MMU in remote areas in France. This population health intervention research is part of a collaborative project for MMU evaluation in reducing or even eliminating territorial inequalities in participation in OBCS in France. The secondary objective is to identify the most efficient modalities for incorporating an MMU in OBCS.

## Methods

### A multi-partner project

This project is collaborative, multi-partner and intersectoral. It involves several research teams, a screening management structure (SMS) (Centre Régional de Coordination des Dépistages des Cancers Normandie), institutional partners, local (departments) stakeholders and associations. It is managed by the U1086 INSERM research team (ANTICIPE), which is in charge of its design and evaluation. The SMS is in charge of organizing the OBCS (collecting data, consent, sending invitations, organization of second reading and managing follow-up). The four French departments where the intervention will take place (Eure, Calvados, Manche, and Seine-Maritime) have purchased the MMU collectively. During the 2 years of the intervention, the MMU will be made available free of charge to the SMS. The SMS oversees its maintenance and recruits the necessary staff (driver, secretary, radiographer and doctors). Doctors in the four departments are partners in the study and devote part of their working time to the MMU. The MMU itinerary is established in collaboration with a geography research team (IDEES UMR CNRS 6266) that will optimize the itinerary by integrating various logistic constraints such as distance and available parking space. Other research collaborations have been established with a social work institute (Institut Regional du Travail Social) to list all medical, social and associative resources that could be mobilized, such as networks of social workers, health centres, medico-social services and associations. These local stakeholders will inform the women about the OBCS and the MMU. Promotion Santé Normandie (health promotion institute) will ensure coordination and common understanding between the partners, and will develop new communication tools dedicated to OBCS in an MMU. The AAPRISS platform (Apprendre et Agir Pour Réduire les Inégalités Sociales de Santé), which is specialized in the social determinants of health and social inequalities in health, will be involved in developing the theoretic basis for the intervention.

The research is funded by the French National Cancer Institute (Institut National du Cancer) for 48 months, by the regional health agency (Agence régionale de Santé Normandie) in charge of OBCS in the region, and by a grant from the association ‘Ruban Rose’ (https://www.cancerdusein.org/).

### Study setting

This 2-year intervention will begin in March 2022 in the departments of Eure, Calvados, Manche, and Seine-Maritime (Normandy Region of France, except the Orne department, which already has an MMU). The territory covered is 23,791 km^2^ with just over 3 million inhabitants.

### Trial design

The design is a prospective randomized controlled cluster trial in the general population with parallel group superiority trial and 1:1 allocation ratio for clusters. The schedule of enrolment, intervention and assessment is shown in Table 1. This protocol is reported according to the recommendations of the Standard Protocol Items: Recommendations for Interventional Trials (SPIRIT).

**Table 1:**
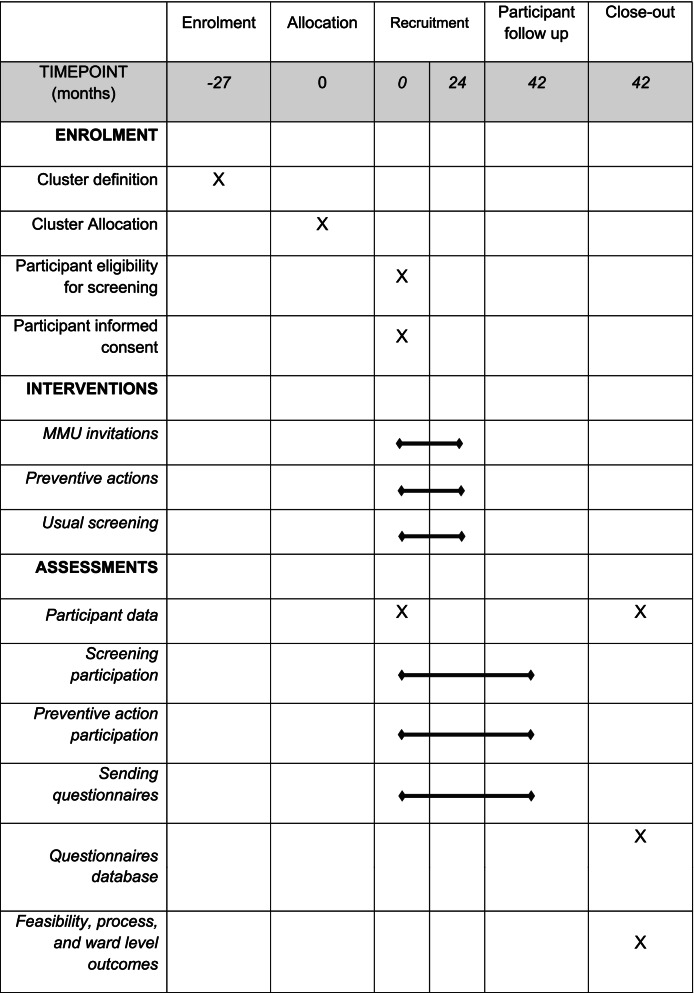
Schedule of enrolment, intervention, and assessment of mobile mammography unit (MMU) trial, following the Standard Protocol Items Recommended for Clinical Trials (SPIRIT) guidelines

### Eligibility criteria

Women invited to the MMU are all women whose last mammogram is more than 22 months old. Compared to the usual cycle of screening campaigns, these are the women invited at the moment when the MMU passes close to them as well as all the women who have not participated since their last invitation.

### Informed consent

In the invitation to screening, a legal notice informs women of the storage and use of their data for evaluation purposes and of their right to object to this use. This trial does not involve collecting biological specimens for storage.

### Intervention

It is now widely accepted that when implementing public health interventions aimed at reducing socio-territorial inequalities, some consensually recognized principles should be adopted. These include the existence of a social gradient across society, the relevance and the value of the principle of proportionate universalism [20] and the multilevel, intersectoral, multidisciplinary nature of the intervention [21]. These principles have been adopted in our program and research.

#### Intervention description: screening appointment

The main intervention consists in an offer of an appointment at the MMU in addition to the current OBCS. This complementary mode of screening offers women who live the furthest away from the radiologist’s office the possibility to undergo screening in the MMU. All women eligible for OBCS and living in the area selected for the intervention are invited by the SMS to participate either in a radiologist’s office or in the MMU. OBCS for women in the control group is not modified. The MMU is equipped with a latest-generation digital scenographer as well as an ultrasound system according to the current rules published by the National Institute of Cancer. The quality of the mammographic radiographic equipment is also certified by the Nuclear Safety Agency. Compliance with the specifications is ensured by the presence on board of the MMU of a radiographer and a doctor. The first reading will be made immediately if the doctor is a radiologist. If necessary, an enlargement, additional images or an ultrasound will be performed. This procedure currently represents between 15% and 20% of all mammographies performed. Otherwise, the first reading is differed and organized with a radiologist subsequently by the SMS. In both cases, when the first reading is negative, the second reading is carried out by another radiologist, as is currently the case.

#### Intervention description: preventive actions

About 2 weeks before the MMU is parked at the appointment site, the women concerned receive information about it. For most women, this is the first time they learn of the existence of the MMU.

The stakeholders provide various forms of information on OBSC and on the organization of screening in the MMU. A dedicated decision-making tool has been developed for this purpose. All those involved have been informed about the need to adhere to the protocol and the importance of respecting the intervention and control groups. Preventive actions are organized in collaboration with the research team and are targeted mainly on the women in the intervention group. However, in the areas where the intervention takes place, preventive actions undertaken prior to the MMU visit are organized by and under the responsibility of the stakeholders according to the territory they cover and their availability. In addition, a dedicated website https://mammobile-normandie.fr/ provides further downloadable information including a video of screening in the MMU and the times and days when it will be parked in their vicinity.

### Assignment of interventions

#### Definition of clusters

The cluster in this study is a group of IRIS (Ilots Regroupés pour l’Information Statistique), which is the smallest administrative unit in France for which census data are available and represents about 2000 inhabitants. Thanks to the geomatics tools of the MapinMed platform at U1086 INSERM (national platform officially labelled by the National League against Cancer), all the women in the target population for OBCS in Normandy are geolocated and geocoded (CNIL authorization N ° 921203). Thus, for each woman, history of screening, date of current screening invitation, IRIS of residence, level of social deprivation according to the European Deprivation Index (EDI) and the distance between the woman’s house and the nearest approved radiologist’s centre are extracted from the SMS database or calculated (EDI, distance). The EDI is an ecological deprivation index constructed according to Townsend’s concept of relative deprivation [8, 22]. It is calculated as the sum of weighted census variables. The 2011 French version of the EDI is computed for each IRIS as 0.32* % country birth + 0.36* % citizenship + 0.21* % tenure status + 0.32* % household size + 2.42* % no bath, no shower + 0.36* % marital status + 0.87* % education + 0.55* % professional activity + 0.80* % months unemployed + 0.70 * % occupation.

#### Sample size calculation

The U1086 INSERM research team has developed an algorithm in python v3.0 to constitute clusters combining some constraints in a regional scenario for the four departments. Eligible population data are available in the SMS screening database. First, it was estimated than the number of intervention days should not exceed 400 over 2 years (considering holidays, climatic and logistical hazards). Eight hundred days were considered to constitute a control arm. Based on the experience of the Department of Orne [10], it was estimated that around a hundred women had to be invited each day in order to perform around 30 mammograms daily. We also plan that, when possible, the MMU will park on the same site for at least two consecutive days so that women will have flexibility in attending. The population study (intervention arm and control arm) has been constituted to be as distant as possible from accredited radiology centres. After calculating the average travel time of women to the nearest radiology centre in each IRIS, 96,200 women were selected (algorithm stopped at the IRIS exceeding maximum population = 800 days of work*120 women = 96,000) in the 1131 most distant IRIS. To maximize aggregation of the IRIS according to travel time to the radiology centres, the most distant ones were selected and then merged with neighbouring IRIS, still by distance travelled, until areas of the expected population size were reached. This algorithm was applied to all IRIS according to distance rank until aggregation was no longer was possible. 91,982 women (95.6%) and 1067 IRIS (94.3%) were selected in the final population in 356 clusters (with 258 created by the algorithm). This algorithm allows geographical clusters to be constructed. The number of women is estimated on the basis of a previous screening campaign, so the real number will be known when the screening invitation has been sent. Table 2 shows the distribution of clusters between departments and arms. As it is a regional calculation according to the criterion of distance, the clusters are not equally distributed among the departments.

**Table 2:** Clusters and distribution of women in each department

Department	Arm	Women number	*N* (%)	Cluster number	*N* (%)
Calvados	Control	14730	26875 (29.27%)	54	102 (27.64%)
	Intervention	12145		48	
Eure	Control	14036	27801 (30.27%)	58	111 (30.08%)
	Intervention	13765		53	
Manche	Control	8022	15630 (17.02%)	30	62 (16.80%)
	Intervention	7608		32	
Seine Maritime	Control	9919	21676 (23.60%)	43	94 (25.47%)
	Intervention	11757		51	
Total		91982		369^a^ (356)	

^a^13 clusters cover two departments

#### Allocation

Randomization in parallel was performed to constitute the intervention arm (*n* = 178 clusters) and the control arm (*n* = 178 clusters), corresponding to 45,275 targeted women in the intervention arm vs 46,707 in the control arm. Randomization was accomplished using a computer-generated random number. The information on the clusters and the intervention and control groups was then recorded in the database of the SMS. Patients, the data manager and the medical staff cannot be blinded regarding the intervention. The main characteristics of each arm are presented in Table 3. The mean travel time is 20.42 min in the intervention arm and 20.48 in the control arm. The higher the deprivation index score, the more the cluster is deprived.

**Table 3:** Characteristics of intervention and control arms

	Intervention	Control
Clusters (*N*)	178	178
IRIS (*N*)	562	505
Women (*N*)	45275	46707
Travel time (min) mean [min-max]	20.42 [16.2;31.2]	20.48 [16.1; 30.1]
EDI mean [min-max]	− 0.77 [− 6,5 ;7.4]	− 0.87 [− 5.6 ;5.8]

**Fig. 1:**
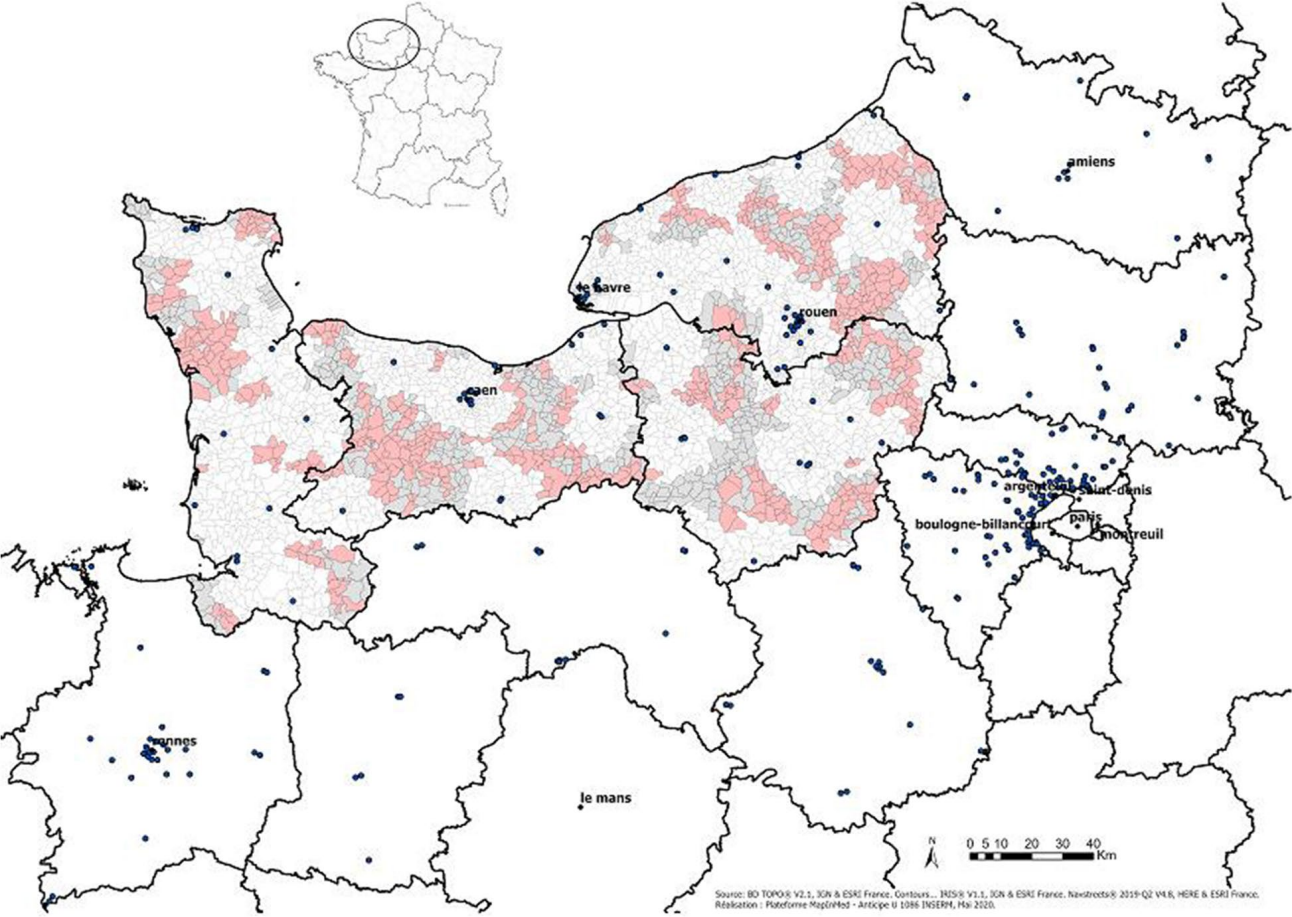
Trial geographic map

Figure 1 represents the map of the four departments concerned by the intervention. Black contours represent the boundaries of the departments and grey contours represent those of the IRIS. The intervention areas are coloured pink and the controls grey. The dots represent the radiology office. The calculation includes the distance to the radiology office of the neighbouring departments.

### Statistical method

The overall objectives of the study are to evaluate the intervention’s ability (a) to increase the participation rate in remote populations, (b) to reduce the socio-territorial inequalities of participation in OBCS in a regional area (Normandy) and (c) to understand how the intervention interacts with contextual factors and which causal mechanisms lead to these results in order to identify the optimal modalities for national rollout. The intervention will be analysed after 2 years.

#### Primary outcomes

Concerning the first two objectives, the main evaluation criterion will be participation in screening. Information on clusters, invitations and participation of women are available in the SMS screening database and recorded according to the quality criteria necessary for the monitoring and evaluation of screening. Participation will be measured and compared between the ‘intervention’ and ‘control’ arms, at aggregate level (cluster) and individual level in intent to treat. In both arms, a woman will be considered a non-participant 18 months after her last invitation. At the aggregate level, the comparison of screening participation will allow us to measure the raw and age-standardized increase in participation due to the intervention. At the individual level, multilevel logistic regressions based on cluster data will allow us to assess the increase in the probability of participating in screening after adjustment on social deprivation, age and other available individual characteristics. The analysis of all the screening data in the study departments, and in particular the comparison of remote (‘intervention’ or ‘control’) and nearby areas, will allow us to assess the impact of the intervention on reducing geographic inequalities in the Normandy region. Although the number of subjects in the study was calculated pragmatically, the intraclass correlation factor was estimated at 0.0083, which with an average area size of 318.6 women gives a design effect equal to 3.63. Therefore, the minimum significant difference in participation that we can prove will be 1.5%. Screening quality criteria will also be monitored using all the data available in the SMS database. Particular attention will be paid to the number of cases of lost to follow-up and delays in reading.

#### Secondary outcomes

In population health intervention research, the effects of an intervention are modified by the characteristics and dynamics of the context in which it is deployed, so including interactions between the intervention and its context will be a methodological challenge [23]. Even if not controlled, factors such as the actions of the stakeholders in prevention will be prospectively registered in a dedicated database and will be included in the model.

Other factors will remain unknown and will contribute to a residual contextual variance which may remain significant even after adjustment for individual variables. Furthermore, to explore contextual effects and to throw light on the causal mechanisms contributing to the effectiveness of the intervention, quantitative analyses will be complemented by a qualitative approach based on approaches that have emerged in realistic randomized controlled trials, using a theory that goes beyond logical models to describe contextual mechanisms and contingencies [24]. An intervention theory is currently being developed and an auto-questionnaire will specifically explore the components of the Com B model (Capacity, Opportunity, Motivation) used by women thanks to the interventions (MMU, preventive action, MMU-specific information tools) [25–27]. A second auto-questionnaire will specifically evaluate their informed choices with the MMU-specific information tools, and a third will probe their level of satisfaction. In order not to call upon the same women several times, we will randomly select three groups of women from the intervention and control arms. Apart from the questionnaire on satisfaction which will be sent only to participating women a few weeks after their participation, the other two questionnaires will be sent to both participants and non-participants.

A medico-economic evaluation will also be performed. A cost-effectiveness analysis of invitation to the MMU (or to a radiologist office) versus invitation to radiologist office (RO) only (usual screening) will thus be conducted from the payer perspective over a period of 2 years, i.e. the duration of a screening campaign. It will determine the efficiency of the invitation to the MMU compared to RO only. The economic evaluation will provide an ICER (incremental cost-effectiveness ratio), which will represent the incremental cost per additional screening of the invitation to MMU compared with the usual screening procedure. A similar approach was already applied for the retrospective analysis of the Orne department MMU [28].

### Governance and monitoring

#### Steering committee

A steering committee was formed at the start of the project with one or two representatives from the main partners. Scientific decisions are made in partnership with the SMS. The frequency of meetings has gradually increased to one per week. The SMS database complies with the regulations in force on the protection of health data. Procedures for the circulation, recording and extraction of data relating to project monitoring have been drafted in order to guarantee the quality and confidentiality of the data.

#### Data monitoring committee

We did not set up a data monitoring committee because what we are evaluating is a new modality for offering breast cancer screening, the MMU, and not the screening act. Protocol modifications will be discussed with partners and funders and if necessary, submitted for ethics committee approval. The trial registration will be updated.

#### Dissemination plans

Results will be published in research publications and conference contributions. Feedback will also be provided to all partners. Scientific reports will be written for funders. The datasets analysed during the current study and statistical code are available from the corresponding author on reasonable request, as is the full protocol.

## Discussion

This population health intervention research involves a wide range of stakeholders who must coordinate their actions according to the conditions and the environment in which they are implemented. The protocol must provide evidence of the effectiveness of the MMU and show that it reduces inequalities in participation in OBCS. At each stage, i.e. protocol, implementation and evaluation, the project must incorporate the existence and the reduction of these inequalities [29].

### Reducing inequalities

The co-construction of this project by teams of researchers, institutional and local stakeholders from different disciplines inside and outside the health field is a real strength since it ensures that the study is comprehensive and multidisciplinary. This will facilitate its implementation and subsequent uptake by all those involved in it. A wide range of professionals from the medical, medico-social, social and associative field will play an active role. Although prevention is the main focus of the intervention, the social and psychosocial dimensions of the subjects will also be considered. Our hope is that women will become more receptive to the need for prevention thanks to this campaign.

Nevertheless, it is a challenge to bring all of the stakeholders onboard and focused on a common goal. The project is time-consuming and was already agreed upon 3 years ago. Some aspects of the project are the responsibility of the partners, so the research team leading it does not have full control over the entire project. For example, the MMU is purchased by the four departments involved in the project, so all the administrative stages of the purchase have to be validated by several levels of authority. However, the SMS is the central partner because of its key role in organizing OBCS, even in remote areas, and women largely understand this. The SMS also undertakes preventive actions in areas where the participation rate is low, and it has established a network of partners to offset this weakness. This network will continue to assist in the operation of the MMU at the end of the two years of the intervention. Thereafter, the MMU will continue to function outside of the constraints of the protocol. Another challenge will then be to incorporate other disciplines such as geography and social psychology into the project. This will be difficult but necessary: on the one hand, different disciplines use different research tools and semantic registers; on the other, fully evidence-based conclusions can be drawn only by including the input of research domains that throw fresh light onto issues that the medical community is unable to investigate alone.

The project is based on consensual models of the social determinants of health, which take individual behaviours and characteristics (proximal determinants) into account and the living environment (distal determinants or causes of causes) in a causal model of inequalities in health [30]. In the typology of interventions to reduce the social inequalities stemming from the dynamic model of the WHO, this is a multilevel intervention operating at access to service, community and individual level. By factoring the MMU into the current breast screening campaign, our design will meet the requirements of the principle of proportionate universalism by providing an additional screening modality for women living far from a radiologist’s office, thus empowering them and reducing the inequalities from which they suffer. In turn, the community in the wider sense will become empowered. Finally producing information adapted to the target population and based on the literacy principle will improve women’s knowledge about OBCS.

### Design

This randomized cluster trial will provide the highest level of evidence to date regarding the effectiveness of an MMU in enhancing the OBCS participation rate in remote areas. The approach is territorial based on administrative units, the intervention targets women who live far from radiology centres approved for screening, and the clusters are randomized into parallel groups. Other designs such as stepped-wedge and cross-over have been studied, but they are not applicable with an MMU that has to move optimally from one area to another [31]. Nevertheless, parallel group randomization carries a risk of bias. Since the main criterion is the distance to the radiology centre, the zones that are randomized are neighbouring. Consequently, even if only women residing in a given intervention area receive an appointment to attend the MMU, information may cross boundaries between areas. Therefore, this potential bias will be monitored.

Another limitation is that the MMU is dedicated to populations living far from radiology offices. Therefore, even if the value of the intervention is proven to be positive, it will demonstrate the potential for the MMU to reduce territorial inequalities but not necessarily social inequalities. For some deprived women, the obstacle to screening participation may be more social or cultural than simply their physical access to a radiologist. In addition, while remote populations are globally less deprived than urban populations, some individuals living in rural areas may be heavily deprived. For this reason, future work will investigate the impact of deploying the MMU in urban areas.

### Mixed evaluation

While this protocol will assess the potential of using the MMU to reduce territorial inequalities, it also contains contextual factors for identifying the conditions in which it could be rolled out nationwide with maximal efficiency. First, we will gather heterogeneous data on what actions or information campaigns have been carried out before the MMU arrives in a given vicinity, and on who provided the information. In turn, this will demonstrate how that information, and how it is given, can be improved. Although a set of well-intentioned stakeholders interact with each other at present, much of that interaction takes place on a piecemeal basis with insufficient overarching global vision. The planned MMU intervention will partly resolve this issue by mobilizing a network of stakeholders to harmonize sources of knowledge, modify the roles they play, and by adapting them to a set of newly emerging constraints, some of which may be as yet unknown. However, they may include issues pertaining to demography, geography, and even political decisions. Ultimately, the information that this study will generate is likely to be useful not only at national level but also in other European countries.

## Conclusions

Providing conclusive data and new knowledge about the mechanisms of participation in OBCS remains a major public health issue. This article presents the design of a clustered randomized trial to evaluate an MMU used for OBCS to reduce territorial inequalities in a remote area of France. Its overarching objective is to reduce health inequalities. Several contextual factors will need to be taken into consideration in this complex population health intervention, so the analysis of the data must be fine-grained. Much care will be taken from the design stage to the publication of our findings to make sure that the mechanisms underpinning the results and the modalities for transfer to other French or European regions are based on robust evidence.

### Trial status

The published protocol corresponds to the protocol at the start of the intervention. The clusters were defined in October 2019. The first invitations were sent on February 18, 2022, and the first intervention will take place on March 2, 2022. The intervention must take place in 4 departments, for 2 years. With all the hazards of interventional research, we expect an end of intervention for March 2024.

## Data Availability

The datasets analysed during the current study are not publicly available before the end of the analyses of the intervention. Any data required to support the protocol can be supplied on request.

## Abbreviations

AAPRISS: Apprendre et Agir Pour Réduire les Inégalités Sociales de Santé
BC: Breast cancer
CNIL: Commission Nationale de l'Informatique et des Libertés
EDI: European Deprivation Index
ICER: Incremental Cost-Effectiveness Ratio
IDEES: Identité et Différenciation de l*’*Espace, de l*’*Environnement et des Sociétés
INSERM: Institut National de la Santé et de la Recherche Médicale
IRIS: Ilots Regroupés pour l’Information Statistique
IRTS: Institut Regional du Travail Social
MMU: Mobile Mammography Unit
OBCS: Organized Breast Cancer Screening
PSN: Promotion Santé Normandie
RCT: Randomized Cluster Trial
RO: Radiologist Office
SMS: Screening Managing Structure
WHO: World Health Organization
